# Commentary: Loin Pain Hematuria Syndrome

**DOI:** 10.29245/2572-9411/2018/4.1169

**Published:** 2018

**Authors:** Natalie M. Bath, Daniel H. Williams, Hans W. Sollinger, Robert R. Redfield

**Affiliations:** 1 Division of Transplant Surgery, University of Wisconsin School of Medicine and Public Health, Madison, WI, USA; 2 Department of Urology, University of Wisconsin School of Medicine and Public Health, Madison, WI, USA

**Keywords:** Loin Pain Hematuria Syndrome (LPHS), Loin pain, Hematuria, Renal autotransplantation

## Abstract

Loin Pain Hematuria Syndrome (LPHS) remains a rare disease but has a significant impact on those affected by it. Patients diagnosed with LPHS experience severe, constant or intermittent flank pain that radiates to the groin and may be exacerbated even by a gentle touch. These patients often require significant narcotic regimens for pain control and are unable to maintain a functional lifestyle. Previously, diagnosis has been made based on clinical presentation. One treatment for this syndrome is renal autotransplant; however, success rates are varied. Therefore, patient selection for this procedure is important. We have developed the UW-LPHS test as a diagnostic maneuver in order to determine which patients with LPHS would benefit from renal autotransplant. To perform this diagnostic test, bupivacaine is instilled into the ureter on the affected side and left to dwell. Patients who experience pain relief following this test are deemed to benefit from renal autotransplant. Here we describe this novel diagnostic test and initial success rates following renal autotransplant.

Loin pain hematuria syndrome (LPHS) describes a constellation of symptoms that is estimated to have a prevalence of approximately 0.012% and primarily occurs in women.^1^ The most significant symptom that patients experience is severe flank (loin) pain that may be unilateral or bilateral and radiates to the abdomen, medial thigh or groin.^2–4^ Pain may be intermittent or constant but has previously been described as “possibly the worst pain known in medicine” and can be exacerbated by common daily activities such as exercise or riding in a car. As a result of this debilitating pain, patients often require large quantities of narcotics for pain control.^5^ Additionally, patients may experience micro- or macroscopic hematuria. LPHS has previously been differentiated as type 1 or type 2 LPHS. Type 1 LPHS can be attributed to identifiable causes including nutcracker syndrome, nephrolithiasis, polycystic kidney disease, recurrent renal papillary necrosis with ureteral obstruction, renal thromboembolism, or renal artery dissection. Cases in which diagnostic work-up does not reveal an etiology have been categorized as type 2 LPHS.^5^ Although the pathophysiology of type 2 LPHS remains largely undetermined, Taba Taba Vakili and colleagues describe one possibility that postulates that the pain and hematuria seen in LPHS may be related to renal vascular disease, coagulopathy, and renal vasospasm with microinfarction among other abnormalities in the urogenital system.^3,6–8^ However, renal function is usually unchanged in these patients.^9–11^ As a result of the fact that pathology cannot be established in a subset of patients with LPHS, these patients are often labeled as having a somatoform pain disorder or drug-seeking behavior.^5,12^

Taba Taba Vakili and colleagues in their 2014 review article *Loin Pain Hematuria Syndrome* describe the pathophysiology, diagnosis, and treatment of this disease. Overwhelmingly, the diagnosis of LPHS was made clinically with the findings of severe flank pain, hematuria, and an exclusion of other causes of these symptoms.^2^ Additionally, this review includes an overview of treatment options and associated success rates including renal denervation, unilateral nephrectomy, and renal autotransplant.^1,11,13–19^ Specifically, they address renal autotranplant as a potential treatment option; however, pain recurrence varied greatly as indicated in multiple case series. One potential explanation for varied success rates following renal autotransplant is improper patient selection. Therefore, it is imperative to have a reliable diagnostic test in order to determine who may benefit from renal autotransplant.

A case report in 2015 described a male patient who presented with symptoms consistent with LPHS. After receiving tadalafil (Cialis, Lilly USA, Indianapolis, IN, USA), which functions as a smooth muscle relaxant of the urogenital system, the patient experienced a dramatic improvement in his pain.^20^ This case highlights the fact that ultimately, the ureter may be the origin of pain in LPHS. To support this hypothesis, we developed the UW-LPHS test as a diagnostic study to identify patients who may experience pain relief following renal autotransplant.

The UW-LPHS test utilizes bupivacaine in order to determine if the ureter is the origin of pain. Under general endotracheal anesthesia, patients undergo cystoscopy and 0.5% bupivacaine is injected into the ureter on the effected side and left to dwell there for 5 minutes. At this time the bladder is also inspected in order to rule out other pathology. Patients are then awoken and their pain is assessed several hours following the procedure before discharge and later in the evening on the same day of procedure. Patients are not given any additional analgesics or relaxants. If patients have relief of their pain, then they are deemed to be candidates for renal autotransplant.^5^ Pain is assessed prior to discharge following the procedure, later in the evening by telephone, and in clinic the following day. Patients who have pain relief for at least 12 hours following the test and have no other anatomical abnormalities are considered to have a positive test and would likely benefit from renal autotransplant. Patients typically state they have pain relief immediately after waking up from general anesthesia, which can then last up to 48 hours following the test.

At our institution, we have utilized the UW-LPHS test in order to identify who may benefit from renal autotransplant for LPHS. Six patients underwent the test and reported significant improvement in their pain. Additionally, two patients did not experience pain relief following bupivacaine infusion. One of these patients was found to have interstitial cystitis, which was diagnosed on cystoscopy, and the other patient was later diagnosed with pudendal neuralgia. The patients who were found to have a positive UW-LPHS test underwent renal autotransplant. The kidney on the affected side is removed with the remaining distal ureter completely removed to the level of the bladder. The kidney is then re-implanted on the right side in standard fashion.^5^

Pre-operatively all six patients required pain medication prior to autotransplant with 50% (n=3) of patients requiring narcotics. Pain medication usage significantly declined at 3 months post-transplant and at present, none of these patients require narcotics. All patients have returned to school or work.

Loin Pain Hematuria Syndrome continues to be not only a rare disease, but also it is incompletely understood. Nevertheless, patients who are affected by it experience life-altering pain, which frequently leads to disability. Although conservative management and surgical intervention have varied results, multiple case series have reported high success rates with renal autotransplant. As reported in Vakili and associates, Burke and Chin presented a series of 48 patients who under renal autotransplant with sustained pain relief in 70% of these patients.^17,21^ However, smaller series have reported pain recurrence up to 73%.^13,14^ We believe one key explanation to this discrepancy in outcomes is patient selection. Since we have identified the ureter as a contributor to pain through ureteral spasm, we have developed the UW-LPHS test as a method to identify patients who may benefit from renal autotransplant. Although additional follow-up is required, our current results indicate that it is a reliable method to identify these patients. As a result, we support the UW-LPHS test as a simple and reliable diagnostic tool in order to help determine the best treatment for patients with LPHS.
